# Mitochondrial Alterations in Prostate Cancer: Roles in Pathobiology and Racial Disparities

**DOI:** 10.3390/ijms24054482

**Published:** 2023-02-24

**Authors:** Kunwar Somesh Vikramdeo, Amod Sharma, Shashi Anand, Sarabjeet Kour Sudan, Seema Singh, Ajay Pratap Singh, Santanu Dasgupta

**Affiliations:** 1 Mitchell Cancer Institute, University of South Alabama, Mobile, AL 36604, USA; 2 Department of Pathology, College of Medicine, University of South Alabama, Mobile, AL 36617, USA; 3 Department of Biochemistry and Molecular Biology, University of South Alabama, Mobile, AL 36688, USA

**Keywords:** prostate cancer, mitochondria, racial disparity, biomarkers, pathobiology

## Abstract

Prostate cancer (PCa) affects millions of men worldwide and is a major cause of cancer-related mortality. Race-associated PCa health disparities are also common and are of both social and clinical concern. Most PCa is diagnosed early due to PSA-based screening, but it fails to discern between indolent and aggressive PCa. Androgen or androgen receptor-targeted therapies are standard care of treatment for locally advanced and metastatic disease, but therapy resistance is common. Mitochondria, the powerhouse of cells, are unique subcellular organelles that have their own genome. A large majority of mitochondrial proteins are, however, nuclear-encoded and imported after cytoplasmic translation. Mitochondrial alterations are common in cancer, including PCa, leading to their altered functions. Aberrant mitochondrial function affects nuclear gene expression in retrograde signaling and promotes tumor-supportive stromal remodeling. In this article, we discuss mitochondrial alterations that have been reported in PCa and review the literature related to their roles in PCa pathobiology, therapy resistance, and racial disparities. We also discuss the translational potential of mitochondrial alterations as prognostic biomarkers and as effective targets for PCa therapy.

## 1. Introduction

Prostate cancer (PCa) is the second most diagnosed cancer in men in the United States, with an expected 268,490 new diagnoses in 2022. It is also the fifth leading cause of cancer-related mortality, with an estimated 34,500 deaths this year [1]. Significant disparities in PCa incidence and health outcomes are reported among various racial and ethnic populations. African American (AA) men bear the highest burden of PCa. They are 1.7 times more likely to be diagnosed with PCa and more than twice more likely to die because of it than Caucasian American (CA) men [2,3]. The underlying causes of such large disparities are not well understood but could involve a variety of factors, including access to quality healthcare, lifestyle, social exposures, and ancestry-related predispositions [4,5].

PCa is a highly heterogeneous disease. Most patients are diagnosed early, especially in developed countries, due to prostate-specific antigen (PSA)-based screening. However, PSA fails to discern between indolent and aggressive PCa and remains a concern for overdiagnosis. Moreover, most positive PSA tests are found to be false positives, thus making it an unreliable biomarker for the prediction of PCa [6], thus warranting a need for the development of newer, specific, and reliable biomarkers. Androgen deprivation therapy (ADT) or castration therapy (CT) is the primary treatment option for patients with locally advanced or metastatic PCa; however, therapeutic failure is inevitable in most patients. Castration-resistant (CR) PCa is highly aggressive and difficult to manage. The use of androgen receptor (AR) targeting agents such as abiraterone and enzalutamide is effective, but therapy resistance develops sooner or later, culminating in patient death [7,8]. Thus, we desperately need newer sets of biomarkers and therapeutic targets to curb PCa-associated mortalities.

Mitochondrial alterations in cancer and their relevance as a biomarker have been re-explored in recent years [9,10,11,12]. Mitochondrial DNA is potentially a better biomarker as its genome is well characterized, and its high copy number allows its alteration to be assessed easily from even a limited amount of samples [13]. Mitochondria, the ‘powerhouse’ of the cell, play a crucial role in cellular metabolism and energy production via oxidative phosphorylation (OXPHOS) [14]. Mitochondria also communicate with the nucleus to convey the information needed to adapt to the metabolic demands of the cell, as well as with the cell’s surroundings leading to stromal remodeling [15]. Here we review the mitochondrial alterations reported in PCa and discuss their roles in pathobiology and racial disparities. We also discuss potential strategies to target dysfunctional mitochondria, as well as their utility as prognostic biomarkers.

## 2. Mitochondrial Alterations in Prostate Cancer

**Figure 1:**
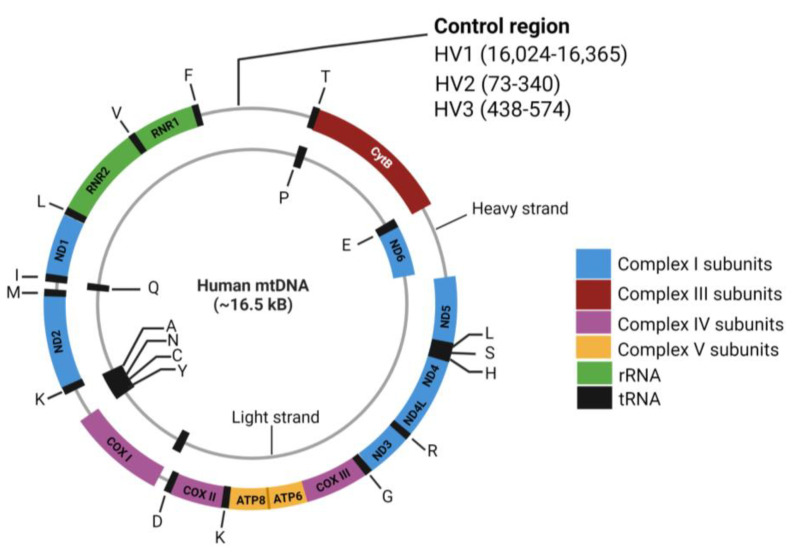
Mitochondrial genome. Mitochondrial respiratory complex function is maintained by both nDNA- and mtDNA-encoded genes. The transcripts of the nDNA-encoded respiratory complex subunits are translated in the cytoplasm and imported into the mitochondria through the mitochondrial import machinery. Thirteen protein-coding genes in mt genome belong to various respiratory complexes (I: *ND1*, *ND2, ND3, ND4, ND4L, ND5*, and *ND6)*; III: *CYTB*, IV: *COXI*, *COXII, COXIII*, and V: *ATP6* and *ATP8*). The noncoding portion of mt genome consists of rRNA and 22 tRNA genes that are important for translation of the mtDNA-encoded transcripts. The control region, sometimes referred to as the D-loop, consists of three hypervariable segments termed HV1, HV2, and HV3. This region also contains replication initiation sites and promoter regions for transcription of the two mtDNA strands. Figure was created with BioRender.com (accessed on 19 February 2023).

Mitochondria contain their own circular genome (16.5 kb) in multiple copies located in the mitochondrial matrix. It consists of genes coding for 13 mitochondrial proteins (subunits of respiratory complexes), 2 ribosomal rRNAs (12s and 16s rRNAs), and 22 transfer RNAs (tRNAs), along with a noncoding region termed as D-loop (Figure 1). The mitochondrial genome lacks protective histones and a robust DNA repair machinery, which makes it particularly susceptible to DNA damage. This vulnerability, coupled with the presence of numerous copies of mitochondria, often causes heteroplasmy, a state where a proportion of mitochondria in the cells have alterations in their mitochondrial genome. Phenotypic changes in the cell often take place when the level of heteroplasmy crosses a threshold and leads to altered mitochondrial function and signaling changes within the cell. A comprehensive tissue analysis of somatic mtDNA alterations in 1675 cancer cases, including 80 cases of PCa, displayed a significant proportion of somatic mtDNA mutations, predominantly single-nucleotide variations, followed by insertions and deletions. Further, this study suggested that functionally detrimental mtDNA mutations are more likely to be heteroplasmic [16]. Below we discuss the various types of aberrations that can potentially alter mitochondrial function.

### 2.1. Mitochondrial Genome Variations

#### 2.1.1. Copy Number Variation

There have been reports that suggest DNA damage usually corresponds with increased mitochondrial content. However, the information in the literature about the variation in mtDNA content in PCa has been quite ambiguous. A high mitochondrial content has been associated with the poor prognosis and aggressiveness of PCa [17,18] and has also been reported to correlate with early-stage PCa patients [19]. In contrast, there have also been studies that link low mtDNA content in the peripheral blood leukocytes to the increased risk of developing an aggressive form of PCa [20,21] and poor prognosis. In a study on PNT1A cells, prostate epithelial cells, which were depleted of mtDNA content by treating them with ethidium bromide, displayed enhanced cell survival and migration through the activation of the PI3K-Akt pathway [22], which seems to corroborate in vitro studies that report that PCa cells with a low mtDNA content show an increase in cell growth and survival. High-grade PCa tumors have been reported to display a higher mitochondrial copy number compared to low-grade PCa tumors [23]. However, a study by Kalsbeek et al. in PCa tissues suggests that the depletion of the mtDNA copy number in PCa tissues is not uniform and rather displays the heterogeneous nature of PCa. They also reported that a high mtDNA content in normal adjacent prostate tissue may be associated with poor prognosis [24]. PCa cell lines with low mtDNA display androgen independence and thus promote therapy resistance [25].

#### 2.1.2. Mutations

##### Mutation in Mitochondrial-Encoded Genes

Mutations in the mitochondrial genome are associated with the initiation and progression of a variety of cancers. The association between alterations in the mitochondrial genome and PCa has been known for quite some time now. Mitochondrial point mutations and mtDNA instability are known to occur at a high frequency in PCa [26]. In fact, in a study performed with 64 tumor samples from 55 PCa patients, the mitochondrial genome displayed a 55-fold higher mutation rate compared to the nuclear genome [27]. In another study involving the next-generation sequencing of 115 PCa tumor samples, 74 unique PCa-specific somatic mtDNA mutations were identified. Most of these mutations were single-nucleotide variants (SNVs) and correlated with disease relapse [24]. A study identified a high frequency of 309 C-T mutations in the D-loop of mtDNA of PCa patients [28]. Another study carried out on 384 tumors from PCa patients reported HV1, a part of the D-loop, as the most frequently mutated region. They also identified 157 SNVs in the protein-coding regions and observed that *MT-ND5* was the most frequently mutated respiratory complex subunit, followed by *MT-ND1* and *MT-CO1* [29]. Earlier studies have associated the mutation of the respiratory complex to be predominant in PCa patients. Mutations in *MT-ND2* and *MT-ND4* have been linked to early-stage PCa [30,31]. Mutations in other respiratory complexes, such as MT-CYTB (A14769G) and *MT-ATP6* (C8932), have also been reported to be associated with PCa and promote the growth of PCa cells [32]. Further, T8993G mutations in the *MT-ATP6* gene have been shown to promote PCa cell growth and invasion in the bone stromal environment by the modulation of FGF-1 and FAK expression in mice [33].

##### Mutation in Nuclear-Encoded Mitochondrial Genes

In addition to the alteration of the mitochondrial genome, changes in nuclear-encoded mitochondrial genes have also been linked to PCa. Several studies have reported mutations in TCA-cycle enzymes such as fumarate hydratase and isocitrate dehydrogenase in PCa [34,35,36]. One study reported two *IDH1* mutations, i.e., R132C and R132H, to be prevalent in PCa, although these did not correlate with either the stage or grade of PCa [37]. A subsequent study further seemed to confirm the occurrence of these two R132C mutations of *IDH1* in PCa [38].

### 2.2. Alteration of Mitochondrial Respiratory Complexes

Most of the mutations in the mtDNA reside in the respiratory complexes and as such have the potential to effect significant changes in mitochondrial functions and metabolism. There has been increasingly accumulating evidence in the literature underlining the important role of OXPHOS in the progression and development of several types of cancers. As such, there is scant evidence in the literature about changes in metabolism and their significance in the development of PCa. Drug-resistant PCa cells have been shown to primarily depend upon OXPHOS rather than glycolysis [33]. In addition, drug-resistant PCa shows an increased flux of primary fuel sources such as glucose, glutamine, and lactate through OXPHOS [39].

**Figure 2:**
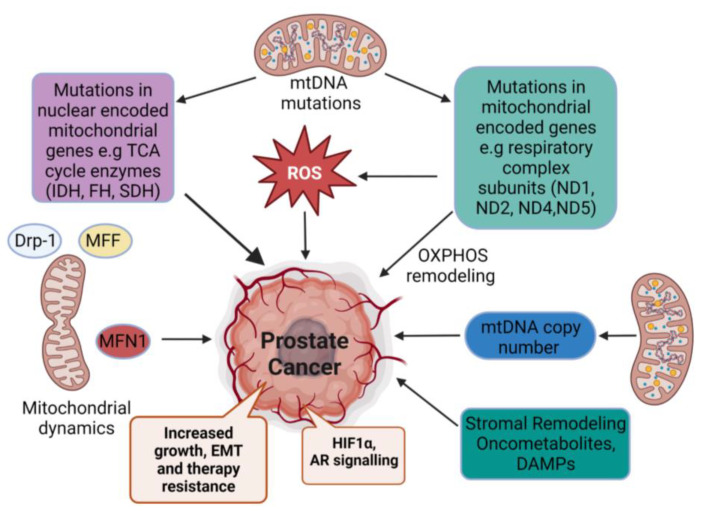
Mitochondrial alterations in prostate cancer and its causative factors. Mitochondrial alterations comprise mutations in mtDNA, which can lead to enhanced reactive oxygen species (ROS) production and promote cancer growth and survival. In addition, reprogramming of TCA-cycle metabolites such as citrate and succinate in conjunction with alterations in copy number enable cancer cells to increase their energy production. Changes in mitochondrial dynamics reshape mitochondrial metabolism and play an important role in PCa growth, EMT, and therapy resistance. Modulation of HIF1α and AR signaling, along with stromal remodeling and oncometabolites, play an important role in progression of PCa. Figure was created with BioRender.com (accessed on 15 February 2023).

The expression of mitochondrial respiratory complexes has been linked to early-onset PCa. Some earlier studies have reported the reduced expression of *MT-RNR1, MT-CO2*, and *MT-ATP6* in PCa tumor samples [40,41]. In a study carried out by Verma et al. in the transgenic adenocarcinoma of a mouse prostate (TRAMP) model, they showed the reduced expression of nuclear-encoded mitochondrial genes such as COX10, COX15, and *COX17,* along with *MT-ND4, MT-CO1, MT-CO2*, and *MT-CO3* [42]. Another study reported the reduced expression of *NDUFS4, SDHA, UQCR2, MT-CO1*, and *ATP5F1A* in tumor samples from 94 PCa patients who had undergone radical prostatectomy after tumor diagnosis. The most depleted subunit reported in this study was ATP synthase F1 subunit alpha (ATP5F1A) [43]. Although earlier studies have reported alterations in Complex I in PCa, increasing evidence suggests a pivotal role of Complex II in the promotion of PCa tumorigenesis. The cBioPortal database analysis shows several alterations in SDHA and SDHB genes in PCa patient samples [44,45]. Recent reports show that PCa cells preferentially utilize succinate oxidation for their metabolic needs [46,47]. A recent study by Schopf et al. suggests a link between mtDNA mutations and shifts in metabolism in the context of the substrate used for energy production in PCa. They report that benign and normal prostate tissues display a higher dependency on glutamate- and malate-driven OXPHOS, while malignant tissues primarily depend upon the oxidation of succinate for their energy [23]. The most frequent mutation of protein-coding genes was the T10551C mutation in the *MT-ND4L* gene. This study also provides evidence that mutations in respiratory complexes display an optimal shift in the metabolism at heteroplasmy levels of around 30–60%. Further, it suggests that while alterations in Complex IV can adversely affect the total OXPHOS capacity of the cells, alterations in Complex I can be sufficiently compensated. A reduction in the expression of NADH–ubiquinone oxidoreductase subunit B8 (NDUFB8), an accessory subunit of Complex I, is reported to be critical for Complex 1 assembly and function [48]. A high-throughput analysis of formalin-fixed PCa tissue samples revealed that malignant tissue displayed a reduced expression of *NDUFB8* and *MT-CO1.* These tissues also revealed a high mitochondrial mass, which may suggest a potential compensatory measure by the cell to cope with respiratory complex dysfunction [49]. Altogether, these mitochondrial variations lead to many diseases, including cancer (Figure 2).

### 2.3. Alteration of Mitochondrial Regulatory Factors

#### 2.3.1. Mitochondrial ROS

ROS has been widely known to aid in the neoplastic transformation and aberrant growth and proliferation of cells [50]. Changes in the ROS levels of the cells trigger the activation of a variety of signaling pathways that contribute to cell survival under oxidative stress conditions [51]. These processes are reported to be responsible for the initiation and progression of many cancers, including PCa [52]. Several studies over the last few years have established a crucial role of oxidative stress in the development of PCa [36,53,54]. Tumor cells inevitably create a hypoxic environment as a consequence of their rapid and unchecked proliferation. To counteract the consequences of a low oxygen environment, cancer cells stabilize and activate hypoxia-inducible factor (HIF-1) [55]. However, it has been shown that this also results in an increase in ROS generation, with the predominant source being mitochondria. When PCa cells are exposed to a hypoxic environment, the modulation of ROS levels along with metabolism promotes its survival and growth [56]. High ROS levels can further sustain the expression of HIF-1 by inhibiting prolyl hydroxylases, which usually degrade HIF1. ROS can also promote the formation of new blood vessels by increasing the expression of VEGF [57]. Mitochondrial glycerophosphate dehydrogenase (mGPDH) increases ROS generation in PCa cells and sustains elevated glycolysis [58].

#### 2.3.2. Antioxidants

Cancer cells keep a delicate balance of ROS to maintain its growth-promoting potential while at the same time avoiding its cytotoxic effects. To achieve this, they often rely on altering the expression of antioxidant genes. Erythroid 2p45 (NF-E2)-related factor 2 (Nrf2), a master regulator of the antioxidant-response system, carries out its function by binding to the antioxidant-response element (ARE) present in various antioxidant genes [59]. The loss of Nrf2 expression has been shown to occur in PCa, and further studies in a knockout mice model show that it results in a reduction in GST levels, enhances ROS, and correlates positively with PCa development [60]. The restoration of Nrf2 levels has been reported to cause a reduction in the anchorage-independent growth of PCa cells [61]. However, some studies have reported that high Nrf2 levels are beneficial in countering the proteotoxic stress in PCa cells and may be involved in enhancing its aggressiveness [62]. Furthermore, Nrf2 can also promote chemoresistance by the maintenance of cancer stem cells [63].

#### 2.3.3. PGC1α

Peroxisome proliferator-activated receptor gamma coactivator 1 (PGC1) is a family of transcriptional coactivators that are important for the regulation of mitochondrial biogenesis. Recent studies have shown that PGC1 is downregulated in PCa patients, which increases the migration and invasion of PCa cells [64]. Further, PGC1α expression negatively correlates with the Gleason score [65]. Its expression decreases as the disease progresses towards the metastatic state. The restoration of PGC1α expression inhibits growth and metastasis in PCa cell lines [65].

#### 2.3.4. Androgen Receptor Signaling

AR can promote the expression and activity of IDH1, a key enzyme of the TCA cycle, and thus reprogram the metabolism of PCa cells [66]. AR also possess a mitochondrial localization signal (MLS) and is shown to be localized inside mitochondria in both PCa tissues and cell lines [67]. AR signaling also causes the increased production of TCA-cycle enzymes and intermediates such as citrate synthase, acetyl-CoA, and oxaloacetic acid and leads to castration resistance [68]. AR also upregulates DRp-1, a protein integral to mitochondrial fission, which then helps in the formation of the VDAC-MPC2 complex, which facilitates enhanced pyruvate transport into mitochondria and increases OXPHOS [69].

#### 2.3.5. Heat-Shock Proteins

One of the most important heat-shock proteins in mitochondria is Hsp60, which is crucial for maintaining protein homeostasis in mitochondria. HSP-60 along with HSP-27 has been suggested as a potential biomarker for PCa recurrence [70]. A high expression of HSP-60 has been associated with poorly differentiated PCa and reduced survival. HSP-60 interacts with caseinolytic protease P (ClpP), a mitochondrial protease responsible for degrading unfolded or misfolded proteins in mitochondria and promoting cell survival. This interaction has been shown to promote the growth of PCa cells [71].

## 3. Impact of Mitochondrial Alterations on Prostate Tumor Cell Phenotypes

### 3.1. Role in Prostate Tumor Cell Growth, Aggressiveness, and Epithelial-to-Mesenchymal Transition

The literature suggests that PCa progression is fraught with an increase in ROS that promotes its aggressiveness. During the transformation and later stages of PCa development, PCa cells increase their mitochondrial respiration along with a high glycolytic rate to meet their energy requirement. As a result of enhanced mitochondrial respiration, ROS levels rise, inducing the signaling pathways associated with PCa growth and survival [72,73,74]. Further, advanced stages of PCa are marked by the elevation of the TCA cycle and increasing levels of citrate, which are utilized by the cancer cells for biomolecule synthesis to support their growth [68]. The epithelial-to-mesenchymal transition (EMT) provides cancer cells with an enhanced migratory and invasive capacity, facilitating tumor dissemination and metastasis. Transcription factors involved in EMT also orchestrate intricate metabolic reprogramming that fulfills the increased energy requirement created by a high motility and growth rate [75]. Reports have shown that oncogenic mutations in mitochondrial metabolic enzymes, succinate dehydrogenase, fumarate hydratase, and isocitrate dehydrogenase induce EMT in cancer cells [76,77,78]. Mutations in isocitrate dehydrogenase isoforms IDH1/2 were found in several cancers, including PCa [79]. The mutant isocitrate dehydrogenase enzyme can produce 2-hydroxyglutarate from α-ketoglutarate, which has been shown to act as a potent oncometabolite inducing EMT in several cancers [80,81,82]. The accumulation of oncometabolites because of mutations in mitochondrial enzymes causes epigenetic changes by affecting chromatin structure and function and influencing the signaling pathways involved in EMT [83,84]. In some reports, the downregulation of mitochondrial proteins involved in OXPHOS has also been shown to correlate with increased EMT and aggressive disease features [85,86].

### 3.2. Role in Therapy Resistance

Androgen deprivation therapy (ADT) is a first-line therapy against PCa. However, in the majority of cases, the patients develop resistance and stop responding to ADT, a phenomenon called castration resistance. The development of castration resistance is marked by the switching of glycolytic metabolism to OXPHOS [33]. mtDNA mutations are known to alter the response of PCa cells to chemotherapeutic agents. A mutation in *MT-CO2* (m.6124CT>C) was reported to impair the sensitivity of PCa cells to statin treatment [87]. Changes in the expression of mitochondrial genes also correlate with PCa growth, survival, and resistance. Mitochondrial fission factor (MFF) and dynamin-related protein-1 (Drp1) are also reported to be amplified in castration-resistant PCa and lead to poor patient survival [88]. MFF is also shown to be implicated in the maintenance of PCa stem cells, which further reiterates its importance in the promotion of castration resistance [89]. In a recent study, it was observed that PCa cells secrete mtDNA, which in turn causes the production of C3a, an anaphylatoxin, which then promotes resistance to docetaxel and tumor progression [90]. Ceramides are produced in the ER and transferred to mitochondria via mitochondria-associated membranes (MAMs) and play an important role in programmed cell death (apoptosis), cell cycle, and differentiation [91]. Ceramides are reported to be crucial in the development of resistance to AR inhibitors such as enzalutamide in PCa [92].

### 3.3. Role in Evasion from Apoptosis

Apart from their function as the powerhouse of the cell, mitochondria also play an important role in the cell death pathway such as apoptosis. Mitochondria are crucial for the activation of apoptosis via the intrinsic pathway in response to excessive oxidative stress and DNA damage [93]. Since cancer cells are known to generate excess ROS, they must strive to inhibit the mitochondrial apoptosis apparatus. Bcl-x is a member of the Bcl-2 family of proteins and acts as an anti-apoptotic protein by inhibiting the release of cytochrome c. A higher expression of Bcl-x is associated with high-grade PCa tumors along with both lymph node and distant metastasis [94,95]. Sirtuin 4 (SIRT4) is a mitochondrial matrix protein and has been shown to halt cell proliferation by inhibiting glutamine metabolism in response to DNA damage [96]. A recent study has shown that SIRT4 is degraded via ubiquitination, promoted by the action of p21-activated kinase 6 (PAK6) [97]. Interestingly, the expression of PAK6 is known to be elevated in PCa [98,99], suggesting its important role in the promotion of the cell survival of PCa cells. Trefoil factor 3 (TFF3), a secretory product of mucin-producing cells, is overexpressed in PCa, and promotes cell survival by inhibiting mitochondria-dependent apoptosis [100,101].

## 4. Mitochondrial Alterations in Stromal Remodeling

The high growth rate of cancer cells creates a higher requirement for energy metabolism and cellular building blocks. Cancer cells use various strategies to obtain and utilize nutrients for their survival, growth, and metastasis. PCa development and progression are impacted by rewiring of the mitochondrial metabolism and mitochondrial adaptation. mtDNA mutations in PCa resulted in OXPHOS remodeling and increased succinate oxidation [23]. Cancer cells are known to modulate mitochondrial function in the surrounding stromal cells for the supply of high-energy metabolites. PCa cells alter the mitochondrial metabolism in stromal cancer-associated fibroblast (CAF) cells and create a nanotube to transfer mitochondria from CAF cells to cancer cells [39]. Cancer cells use mitochondrial metabolites and signaling pathways to remodulate their stromal composition and metabolism, which provides a positive microenvironment for tumor growth.

### 4.1. Mitochondrial Damage-Associated Molecular Patterns

Damage-associated molecular patterns (DAMPs) are a large number of chemically unrelated molecules that are retained in normal living cells and during cell death or stress and are released, causing a strong induction of sterile inflammation [102]. Immune cells possess specific DAMP receptors that allow them to sense and react to damage [103]. Research has shown DAMPs could play a crucial role in cancer development and in the host response to cancer therapy. The release of DAMPs from dying cancer could activate the protective function of immune cells, triggering the immunogenic death of the cancer cells. On the other hand, DAMPs could induce chronic inflammation in the tumor microenvironment (TME) and may cause the development and promotion of cancer [104,105]. Typically, DAMPs include extracellular DNA, high-mobility group box-1 (HMGB-1) [106], heat-shock proteins [107], ATP [108], and S100 proteins [108]. S100 proteins act as Ca2+ sensors inside the cells; however, they are secreted extracellularly under stress conditions, can influence a variety of biological processes, and have been reported to be dysregulated in PCa cells [109,110,111]. Mitochondrial DAMPs include mtDNA, ATP released from damaged mitochondrial, N-formyl peptides, succinate, cardiolipin, and cytochrome c [112]. Elevated levels of circulating mtDNA have been found in various cancer types, including PCa [113,114,115,116,117]. mtDNA can be recognized by pattern recognition receptors such as TLR9, type I interferon response, and cytosolic inflammasomes of the innate immune system, and this interaction initiates a proinflammatory response [112]. The activation of TLR9 signaling has been shown to promote the growth of PCa cells and correlate with poor prognosis [118,119]. The HMGB1-TLR4/RAGE axis promotes chemoresistance to docetaxel in prostate tumor cells [106]. DAMP-induced inflammation plays a crucial role in recruiting immune cells in the TME and creating a cancer-promoting immunological niche. Taken together, the decreased mitophagy and increased rupture of mitochondria may enable the release of mitochondrial DNA (or mitochondrial proteins) that serve as DAMPs and promote ROS production, which may act as DAMP modifiers to promote cancer.

### 4.2. Oncometabolites

The accumulation of metabolites due to debilitated anabolic and catabolic processes is a characteristic of mitochondrial alteration. mtDNA mutations and defects in nuclear-encoded mitochondrial enzymes can result in a deregulated mitochondrial metabolism. The accumulated mitochondrial metabolites could serve as cancer-promoting factors by providing growth advantages. These metabolites are referred to as oncometabolites. The mitochondrial metabolites that are well established as oncometabolites are succinate, 2-hydroxyglutarate, and fumarate. A high amount of these metabolites is produced as a result of oncogenic mutations in succinate dehydrogenase, isocitrate dehydrogenase (IDH), and fumarate hydratase enzymes [120]. Mitochondria also exert a robust impact on chromatin structure via the overproduction of oncometabolite 2-hydroxyglutarate, which induces DNA hypermethylation and causes wide-ranging epigenetic changes to support cancer progression [58,121]. The production of oncometabolite 2-hydroxyglutarate is linked with alterations in the gene expression of TCA-cycle enzymes and is known to inhibit the enzymatic activity of ATP synthase and cytochrome-c oxidase [122]. Alterations in IDH lead to the accumulation of its metabolic byproduct, 2-hydroxyglutarate, and have been reported to promote cell invasion in PCa with a negative or low expression of AR [114]. These findings show that isocitrate dehydrogenase mutations in cancer cells result in the accumulation of 2-hydroxyglutarate, which contributes to the energy metabolism changes contributing to the cancer progression. A decrease in TCA-cycle enzyme fumarate hydrates resulted in an increase in transcription factors Nrf1 and Nrf2 [123]. The transcriptional or mutagenic activation of Nrf2 can contribute to tumorigenesis by managing the high ROS produced in PCa cells [62]. In conclusion, the abundance of oncometabolites created by mutation activation or the oncogene-induced activation of mitochondrial metabolic enzymes can lead to mitochondria dysfunction, ROS production, epigenetic modification, increased EMT, and cancer progression.

## 5. Mitochondria Alteration in Prostate Cancer Racial Disparity

Early studies exploring the connections between PCa and mitochondria identified mutations in *MT-CO1* as a risk factor for PCa development. In addition, mitochondrial alterations also show a distinct association with different ethnic groups in the context of PCa [124]. The AA population tends to harbor polymorphism in *CO1* lineages and therefore carries a risk for the development of PCa [32]. However, mutations in *MT-CO1* have also been found to correlate with PCa in CA men [125]. Although both somatic and germline mutations in *MT-CO-1* depict a predisposition for PCa, the latter poses a considerably higher risk. In a study by Petros et al., mitochondrial cytochrome oxidase subunit I (COMI) germline mutation was reported as an important risk factor for PCadevelopment in African American patients. In the same cohort study, some patients also contained a germline ATP6 mutation [32]. PC cell lines harboring mutations in a T8993G mutation in *MT-ATP6* show enhanced growth and proliferation. A study showed enhanced mitochondrial biogenesis and OXPHOS in AA tumors compared to those from European American (EA) patients [126]. AA tumors also had a higher number of mitochondria than their EA counterparts. Overall changes in mtDNA content have also been observed in AA PCa patients. AA tumors also had a higher number of mitochondria than their EA counterparts. A study conducted on AA patients with PCa reported an enhanced mtDNA in the leukocytes, which correlated with an aggressive form of the disease and poor prognosis [127]. Interestingly, normal prostate tissues of AA men also display low mtDNA content compared to CA men, which suggests a potential predisposition towards PCa development [13]. G10398A mutation in *MT-ND3* has been linked with an increased risk for PCa [25]. Furthermore, the cells with this type of mutation displayed an enhanced Complex I activity. Although mutations in *MT-ND-3* and *MT-ATP6* show a racial disparity between AA and CA populations, they do not show any association with the development of PCa in Mexican–Mestizo men, suggesting that factors specific to AA population may be involved in the increased PCa risk in AA men [128].

In a very recent study, researchers identified a significant racial disparity in the expression of pi class glutathione S transferase (GSTP1), a cellular detoxifying enzyme [129]. This enzyme is highly expressed in basal epithelial cells, while it is epigenetically silenced via hypermethylation in many PCa cases and is considered to be an early event in PCa carcinogenesis. However, this may suggest a possibility of the presence of a distinct molecular subtype of PCa and thus requires further investigations. High expression of GSTP-1 in breast cancer has been reported to result in chemoresistance [129,130,131] and thus high expression of GSTP1 in Black men with PCa may predict a poor response to chemotherapy.

An earlier study identified that PCa tumors from AA men show a high expression of zinc transporters hZIP1 and hZIP2 compared to white men, while it is low in normal prostate [132]. The reduction in zinc levels as a result of a lack of zinc transporters relieves the inhibition of mitochondrial aconitase. This modulates the metabolism of PCa cells towards enhanced citrate oxidation, which fuels their growth [133].

Mitochondria also produce small mitochondrial peptides (MDPs) such as small humanin-like peptide-2 (SHLP2), mitochondrial open reading frames (ORF) of the 12S rRNA type-c (MOTS-c), and humanin through small ORFs [134], which are required for normal mitochondrial function. The overall reduction in the levels of these MDPs is shown to increase the risk for PCa development [135]. While CA patients show reduced plasma levels in MDPs, AA patients had an even lower concentration of these, which suggests that AA men are more susceptible to a high risk of PCa [136].

Mitochondrial gene alterations and their association with racial disparity in PCa are depicted in (Table 1).

## 6. Translational Potential of Mitochondrial Alterations in Prostate Cancer

### 6.1. Mitochondrial Alterations as Prognostic Markers

mtDNA is maternally inherited and does not undergo recombination like nuclear DNA. This often results in the accumulation of characteristic mtDNA SNVs within a population, which show variations in their metabolic profiles accordingly. These subpopulations are termed haplogroups and can be a factor in displaying a predisposition towards the development of various pathologies, including PCa [141,143]. mtDNA haplogroup U and its signature A12308G point mutation in tRNALeu2 are associated with a higher incidence of PCa. Thus, people with haplotype U are at a higher risk predisposed, and this haplotype could serve as a prognostic marker for predicting predisposition towards the development of PCa [144]. Furthermore, the analysis of mtDNA content could itself serve as a prognostic marker, as its alteration has been reported by several groups to be associated with various cancers, including PCa [20,21,145,146,147]. High levels of cytochrome c levels in serum from various cancers, including PCa, have been reported and correlated with an advanced and aggressive form of the disease and suggest its significance as a prognostic marker [125]. Although mutations in respiratory complex genes have been reported in a wide variety of cancers, no particular mutation type has been shown to predict the risk for the development of the disease. However, several studies have reported frequent mutations in *MT-ND5, MT-ND4, MT-CO2, ATP6*, and D-loop in PCa patients [29,148,149,150]. The categorization of mutations in these regions in patients may be an effective way to predict the risk for PCa development.

### 6.2. Therapeutic Targeting of Mitochondrial Function

#### 6.2.1. Targeting Prostate Cancer Metabolism

Unlike normal cells, the reprogramming of metabolism is a key cellular process in cancer cells that is responsible for energy production and the synthesis of new molecules to sustain their potential for indefinite growth and proliferation. Emerging evidence suggests that tumor cells show a dependency on mitochondrial metabolism for their various oncogenic properties, such as proliferation, stemness, and chemoresistance [151,152]. Within the same TME, considering tumor heterogeneity, some cells could have a higher glycolytic rate, and others might have a higher mitochondrial respiration. Castration-resistant PCa cells are known for their dependence on OXPHOS for their energy requirements, and as such, respiratory complex inhibitors show excellent potential for the treatment of PCa. Targeting OXPHOS in PCa has been reported to block autophagy and render them sensitive to chemotherapeutic drugs [73,122]. In a normal prostate cell, the metabolic pathways are uniquely regulated to maintain the secretion of prostatic fluid, a primary function of the prostate gland. AR signaling favors the accumulation of zinc in prostate acinar cells, which inactivates the m-aconitase enzyme of the TCA cycle and leads to the synthesis of a large amount of citrate, which is the main component of prostatic fluid and is required for the healthy function of the prostate gland [153,154]. In prostate adenocarcinoma, zinc accumulation is inhibited due to downregulated zinc transporters; therefore, citrate undergoes oxidation through the TCA cycle and produces anabolic substrates required to promote the growth and proliferation of cancer cells [153,155]. The tumor-suppressive role of zinc transporters was confirmed, and it was shown that the overexpression of zinc transporters in PCa cells inhibited NF-κB activity, thereby reducing their tumorigenic potential [156]. Overall, it seems that citrate oxidation is necessary but not sufficient to transform prostate epithelial cells into prostate adenocarcinoma. For a malignant transformation of PCa, the interaction of cancer cells with other cells in the TME is also essential to provide metabolic substrates (e.g., lactate) to cancer cells, which can be used in anabolic pathways as energy support [157,158]. Further, lactate secreted from CAF cells has been shown to regulate the expression of several genes involved in lipid metabolism, which leads to the accumulation of lipid droplets and affects epigenetic modifications in PCa cells [159]. Indeed, the metabolic phenotype of PCa is primarily lipogenic, unlike other solid tumors, and greatly dependent on OXPHOS [160,161,162]. Aberrant AR signaling has been central to regulating metabolic transformation and anabolic processes to fuel the proliferation and growth of PCa cells [163,164]. More specifically, the AR regulates the expression of several genes involved in key regulatory steps of glucose metabolism, fatty acid synthesis, nucleotides, amino acid metabolism, and polyamine biosynthesis [68]. Therefore, the AR antagonism strategy has been highly efficient, as it can also affect the associated metabolic network; however, in the case of androgen-resistant PCa, androgen-independent AR activation takes place to bypass the AR requirement and become a more aggressive AR-indifferent carcinoma [165]. More recently, it has been shown that the inhibition of AR induces distinct metabolic reprogramming rather than suppressing these metabolic alterations [68,166]. Understanding these distinct metabolic features and their connection with AR signaling could lead to the identification of various metabolic vulnerabilities that can be exploited to devise new anti-PCa therapies.

#### 6.2.2. Targeting Mitochondrial Dynamics

Mitochondria undergo continuous fusion and fission to maintain mitochondrial health and function to meet various cellular demands. In many cancers, the aberrant expression of genes regulating mitochondrial dynamics machinery has been reported, and dysregulated fusion–fission has been linked with cancer progression, chemoresistance, and metastasis [167,168]. Androgen signaling enhances DRP1 expression to promote mitochondrial metabolism, including oxidative phosphorylation and lipogenesis. Targeting DRP1 induced a metabolic stress response and autophagy and reduced the AR-mediated growth of PCa [69]. Recently, Civenni et al. have shown that the silencing of BRD4, a chromatin reader protein, inhibits mitochondrial fission and blocks the self-renewal of PCa stem cells, which leads to the loss of tumorigenic capability [89]. Moreover, a mitochondrial Rho GTPase 2 (MIRO2) involved in mitochondrial localization and dynamics has been found to be overexpressed in metastatic PCa compared to localized tumors. The inhibition of MIRO2 markedly suppressed colony formation and tumor growth in vivo and can be exploited as a therapeutic target [169]. The improved mitochondrial dynamics in PCa cells could promote mitochondrial trafficking and increase tumor cell migration and invasion [170]. Mitofusin-1 (MFN1) and mitofusin-2 (MFN2) were reported to be upregulated in PCa patients, as well in PCa cell lines, while MFN2 was also detected in the circulating exosomes of patients with benign and progressive PCa [30]. This observation provides a potential use for MFN2 as both a prognostic and a therapeutic marker. These findings present a strong rationale to target mitochondrial dynamics as a therapeutic treatment to combat cancer progression.

#### 6.2.3. Targeting Translocases and Solute Transporters of Mitochondria

The transport of proteins, metabolites, solutes, ions, and other soluble factors across the outer and inner membranes is crucial for mitochondrial integrity and proper function. Both the outer and inner mitochondrial membranes encompass specialized translocases or transporters for this vital process. The outer membrane encompasses translocases of outer membrane (TOM or TOM40) complexes and TOM/SAM complexes and the voltage-dependent anion channel (VDAC), whereas translocases of inner membrane (TIM) 23 and TIM22 complexes and mitochondrial carrier family (MCF) or solute carrier family (SLC) proteins are present at the inner mitochondrial membrane [171,172]. The expression of these transporters is essential for mitochondrial metabolism and might be implicated in the growth and proliferation of cancer cells. The role of outer membrane transporters is critical, as these proteins provide the entry point for the translocation of several proteins, and the dysregulation of TOM complexes has been linked to cancer progression.

**Figure 3:**
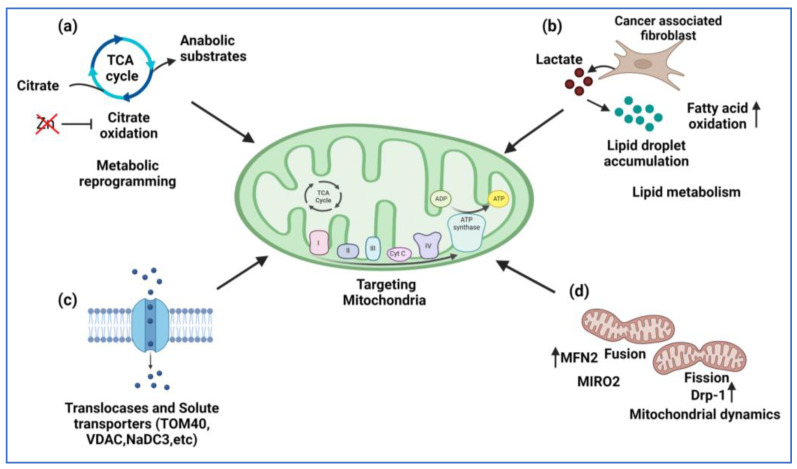
Potential mitochondria targeting strategies in prostate cancer. (**a**) Citrate oxidation is important for transformation of PCa cells, and blocking it could be a first step in treatment of PCa. (**b**) High lactate levels promote interaction between mitochondria and cancer-associated fibroblasts, and thus blocking this interaction would seem to be a viable therapeutic strategy. (**c**) Reducing mitochondrial levels of oncometabolites by altering mitochondrial transporters and (**d**) regulating mitochondrial dynamics via MFN2, DRP-1, and MIRO2 could also provide an effective treatment option for PCa. Figure was created with BioRender.com (accessed on 15 February 2023).

An analysis of multigene signatures identified TOM40 to be altered in PCa patients [173]. Its expression is also upregulated in androgen-independent PCa cell lines and leads to an increase in growth and survival [69,174]. The VDAC is also an important target for cancer therapy due to its role in the transport of glycolytic proteins, protection against apoptosis, and calcium homeostasis [175]. Downregulating the expression of the VDAC in PCa cell lines causes a reduction in their proliferation and tumor growth [176]. The VDAC is also reported to form a complex with mitochondrial fission factor (MFF) in PCa and is important for the maintenance of mitochondrial integrity and function. Hence, this complex can be exploited as a therapeutic option in PCa [88]. PCa cells fuel their OXPHOS via the increased absorption of succinate via the plasma membrane Na^+^-dependent dicarboxylic acid transporter NaDC3 (*SLC13A3* gene). Since this protein is not produced in normal prostate cells, targeting NaDC3 could be a specific and effective target for PCa treatment [177]. Another type of solute carrier family, SLC25 transporters, is the largest family of solute carriers and is involved in the transport of amino acids, cofactors, nucleotides, inorganic ions, protons, fatty acids, and various metabolites associated with the TCA cycle and glycolysis pathways [178]. A bioinformatics analysis of mitochondrial genes using public datasets suggested the differential mRNA expression of SLC25 family members in PCa cell lines [179]. Although these observations require further in-depth studies, SLC25 family members have been implicated in other cancers [180,181] and could turn out to be a potentially effective therapeutic avenue for PCa (Figure 3).

## 7. Conclusions

PCa has a complex pathobiology and is influenced by a variety of factors, such as genetic and epigenetic alterations, environmental factors, and is responsible for the development of the disease. Mitochondria is a very important organelle central to fulfilling the metabolic and energetic demands of the cells, and cancer cells often benefit from their various alterations at every step of tumor development. Since PCa is characterized by heterogeneity in metabolic preferences, the identification and establishment of key mitochondrial alterations associated with PCa could provide us with an excellent noninvasive diagnostic and prognostic strategy for the assessment of normal prostate health and not just tumor malignancy. In addition, the SNVs and mutations of mtDNA may provide us with information about the racial disparity of PCa and could be helpful in devising a precision medicine approach for its treatment. This review summarizes how the various mitochondrial alterations contribute to PCa racial disparity and should direct future studies towards the development of targeted therapeutic strategies that could help in diminishing the racial disparity in clinical outcomes.

## Figures and Tables

**Table 1:** Mitochondrial gene variants and their association with racial disparity in prostate cancer AA—African American, EA—European American, CA—Caucasian American.

S.N.	Gene/Variant	Function	Type of Cancer	Racial Disparity	Reference
1	ERR1 and PGC1α	Mitochondrial biogenesis	Pan cancer	Strong enrichment of ERR1-PGC1α transcriptional program in AA tumors than EA	[126]
2	Cytochrome c oxidase subunit I (COI)	Oxidative phosphorylation, Mitochondrial supercomplex assembly	Prostate cancer	COI missense mutations more common in African American compare to others	[32,137]
3	POLG	Mitochondrial DNA replication	Prostate Cancer	CAG repeats variants in POLG associated with increased risk of breast cancer in African American Women	[138]
4	ND3 (G10398A)	Alters the structure of Complex I	Prostate cancer and Breast Cancer	Increased risk of breast cancer in African American women with G10398A allele	[139,140]
5	Haplogroup U (A11467G, A12308G, G12372A)	-----	Prostate Cancer	Mitochondrial haplogroup U is associated with increased risk of prostate cancer in white North American individuals	[141]
6	HSP60 and HSP90	Act as chaperones and support cancer cell survival	Prostate cancer	Compare to CA cells, lower expression of HSP60 and HSP90 in AA cells is associated with mitochondrial dysfunction, and chemoresistance	[142]

## Data Availability

Not applicable.
